# Letter from Italy

**Published:** 1880-10

**Authors:** A. Lagorio

**Affiliations:** Chiavari, Italy


### Foreign Correspondence.

### ARTICLE V.

*Messrs. Editors:*—The traveler who desires to study the nature and habits of the various populations of Italy, must travel through certain parts of Lombardy, or Venice, and stop to visit the homes of the country people, examining carefully the physiognomy, acts, and words of those who assemble together on festival days at the church of the village. He will thus be astonished by the strange contrast that reigns between the marvelous fecundity of the surrounding country and the misery that is everywhere apparent. The condition of the miserable cottages, the feebleness of the workingmen, their saddened physiognomy, their slow and tired movements, their serious and melancholic conversation distinguish the agricultural population, and show that, in these places, life is truly sad and full of privations. On the other hand if he has traveled through other parts of Italy, an entirely different contrast is presented to his sight. Elegant and commodious dwellings, surrounded by gardens, provided with all the beauties of nature, happy people, healthy, strong, joyous, songful, and playful, present a contrast so great, that he will not fail to ask the occasion of this monstrous anomaly. This notable diversity which for ethnographic reasons ought not to exist, is explained by the nature of the economic conditions to which the people are subjected. The day workman, always the poor servant of the glebe, is poorly lodged, badly clothed and insufficiently remunerated. His labor does not suffice to feed his family, whose spare diet consists wholly of Indian corn meal mush. Every day the organic balance of the poor classes closes with a deficit, and these daily deficits accumulating, result in impoverishment. Little by little the forces fail, all the functions of the body become enfeebled, and the effects of chronic famine are manifest in the terrible *pellagra* which closes the mournful scene. By it the country is depopulated, and the hospitals, the insane asylums, and the cemeteries filled. Italy is the classic country of pellagra. The first study of the disease was made by Dr. Casal, in the year 1735. He called it *rose disease*, from the color that the patient's skin acquires, principally on the dorsum of the hands. A. Pajati studied it about the year 1740 and named it *Alpine scurvy*. In 1740 the pellagra was so much diffused through Italy, that many works on it were written, among them those of Frapolli, physician of the Hospital at Milan. At that time a hospital was founded by Dr. Scambio, exclusively for the treatment of pellagra, wherever this disease prevailed. The extensive cultivation of maize or Indian corn was recognized, and this article was found to be the exclusive diet of that class of inhabitants among whom pellagra became developed. From this it was concluded that Indian corn was the prime cause of the disease. But special conditions are most always present at the crisis of the disease, such as fatigue under the heat of the sun; filthiness of persons and houses; frequent use of acrid oils and irritable vegetables, as garlic and onions; lastly, the scarcity of meats, milk, wines, etc. In 1830, the number of patients affected with this disease in Lombardy alone, amounted to over twenty thousand. In 1856 the number increased to thirty thousand. In 1880 eighty thousand were registered, and in all Italy Dr. Lambroso assures us that no less than five hundred thousand are affected with pellagra. What is to be done to put a stop to this horrible plague? The pauperism of the agricultural class should be remedied in order to remove the cause, and till this is done thousands will fall victims to the pellagra. The Government in this matter can accomplish much.

Some cases of Bright's disease were brought to the clinic this year. In six, the course of the disease has been followed with exactness, noting the daily quantity of urine and albumen, the other principal phenomena and the influence of the various methods of treatment. Special methods were tried, and some of the remedies being quite new, deserve attention, more especially as regards their effects, since the treatment of Bright's disease often improved the condition of the patient and arrested the morbid process. Chronic Bright's disease, untreated, generally does not improve ; therefore it must be excluded from the category of those diseases that sometimes present spontaneous relief. It was noted by Prof. DeRenzi that the patients in the first days after their entrance to the clinic, or when the treatment is interrupted, readily secrete a greater quantity of urine. But there are some exceptions. Fuchsine, recently introduced in the treatment of Bright's disease, produced a sensible diminution of albumen. In clinic it was used under two forms, diluted with water and added to an extract in pillular mass, each of two and one-half centigr. As the intense coloring of the water with fuchsine is objectionable, the pill form is to be preferred. The daily dose of fuchsine may be far greater than that thus far advocated in the treatment of the disease. Ordinarily a small dose of five centigr. was gradually increased to twenty-five centigr. in twenty-four hours. No remarkable physiological action of the fuchsine on the principal functions of the organism has been discovered. According to the dose, the urine commences to present a more or less reddish tint, persisting during all the time of treatment. Generally the latter acquires such a tint about five days after administering the remedy, and loses it about three to five days after suspending the fuchsine. Very often in Bright's disease the urine shows some mucus, and then the fuchsine is of great benefit, the mucus disappearing from the urine. The mucous membranes of the digestive tract become intensely colored by the drug, and the plasma of the blood shows a well-marked coloring. The quantity of hæmoglobin in the blood, and the chromometric grade were examined with the apparatus of Bizzozero, and it became evident that the chromometric grade corresponded to a quantity of coloring matter in the sanguineous plasma, which far surpassed the proportion of hæmoglobin. Evidently, therefore, the more intense coloring is not due to an augment of hæmoglobin, but rather to the dissolution of the fuchsine in the blood. Many consecutive observations have confirmed, according to Prof. DeRenzi, the efficacy of fuchsine as a remedy for Bright's disease. In exceptional cases, where fuchsine does not avail, its presence in the urine is wanting, and the red coloring notably absent. Therefore, when, after some days of treatment with the fuchsine, the urine does not become red, the existence of some obscure alterations of the kidneys must be admitted. Besides fuchsine, other methods have been used, mainly repose in bed, as efficacious means of diminishing the increase of albumen, and to this is added the milk diet. Apomorphine has been generally well tolerated and prescribed in higher doses than those ordinarily given, five to six centigr. daily without the least disturbance. In one case under this remedy the patient's state ameliorated considerably. As a therapeutical remedy the Professor had used the hæmoglobin prepared by Grinon, according to the process of Dr. Lebou, for the treatment of globular anæmia and the absence of hæmoglobin in the blood. So far the observations have been insufficient; but in one case the red blood corpuscles had considerably increased after the use of the hæmoglobin.

Piperine and oil of *eucalyptus globulus* have been largely tried for intermittent fever and tumor of the spleen. The former was largely advocated by Celsus, Dioscorides, Müller, Franck, Altdœfer, Riedmiller, Pettagua, etc. The action of the latter has also been studied by Weber, Gubler, Ullersperger, Tristany, Lambert, Lovinser, Kesser, Mossler, and Gimbert. But the observations in clinic did not furnish such good results as those lauded by these authors, having been found far inferior to those of quinine. As a great number of practitioners attributed either to the piperine, or to the eucalpytus taken separately, a strong anti-febrile action, the two remedies were used together. The formula of Mossler was preferred, that recommended for the the treatment of leukæmia, that is both remedies combined in pill form, each pill consisting of one drop of oil of eucalyptus and four centigr. of piperine. The efficacy of the eucalyptus associated with the piperine was soon confirmed, but yet did not equal that of quinine. The results were good, however, and the Professor did not hesitate to admit that the two combined remedies, eucalyptus and piperine, were the most potent articles next to quinine.

Just at this moment of closing, my eye has fallen on the following in the "Caffaro," of Genoa: "Naples, 11th. Yesterday in Fontana dei Serpi, on Pendino Street, a woman, called *Concetta la Pavattiera*, gave birth to six little creatures, four dead, and two living!" A. LAGORIO, M.D.

CHIAVARI, Italy, Aug. 14, 1880.

